# Supplementary material for: An open-source framework for large-scale, flexible evaluation of biomedical text mining systems
Source: J Biomed Discov Collab. 2008 Jan 29;3:1. doi: 10.1186/1747-5333-3-1 (PMC2276192; doi:10.1186/1747-5333-3-1)
Supplement: Additional file 1 — Evaluation platform source code. The additional file contains the software evaluation framework discussed in this paper. The most current release can be downloaded from SourceForge.net [1]. See the accompanying README file for instructions on installation and use. Also included in the distribution is a collection of UIMA wrappers for some commonly used BioNLP tools and annotated corpora. [file 1747-5333-3-1-S1.zip › bionlp_uima_repository-1.0.1/docs/api/src-html/edu/uchsc/ccp/util/nlp/mention/SlotMention.html]

```
001    /*
002     *
003     * The Original Code is UCHSC Utilities.
004     *
005     * The Initial Developer of the Original Code is University of Colorado.  
006     * Copyright (C) 2005.  All Rights Reserved.
007     *
008     * UCHSC Utilities was developed by the Center for Computational Pharmacology
009     * (http://compbio.uchsc.edu) at the University of Colorado Health 
010     *  Sciences Center School of Medicine with support from the National 
011     *  Library of Medicine.  
012     *
013     *
014     * Contributor(s):
015     *   William A. Baumgartner <william.baumgartner@uchsc.edu> (Original Author)
016     *   
017     * Created: November 2005
018     * Dependencies:
019     * TODO:    This class should allow for multiple slot values 
020     * Changes:
021     * 04/30/06 - wab - added getSingleLineRepresentation()
022     */
023    
024    package edu.uchsc.ccp.util.nlp.mention;
025    
026    import java.io.PrintStream;
027    import java.util.ArrayList;
028    import java.util.List;
029    
030    import edu.uchsc.ccp.util.nlp.annotation.comparison.SpanComparator;
031    import edu.uchsc.ccp.util.nlp.mention.comparison.IdenticalSlotMentionComparator;
032    import edu.uchsc.ccp.util.nlp.mention.comparison.SlotMentionComparator;
033    
034    public class SlotMention extends Mention {
035    
036        private List<Object> slotValues;
037    
038        @Deprecated
039        public SlotMention() {
040            super();
041            slotValues = new ArrayList<Object>();
042        }
043    
044        public SlotMention(String mentionName) {
045            super(mentionName);
046            slotValues = new ArrayList<Object>();
047        }
048    
049        /**
050         * Get the slot values
051         * 
052         * @return
053         */
054        public List<Object> getSlotValues() {
055            return slotValues;
056        }
057    
058        /**
059         * Set the slot values
060         * 
061         * @param slotValues
062         */
063        public void setSlotValues(List<Object> slotValues) {
064            this.slotValues = slotValues;
065        }
066    
067        /**
068         * Return the first slot value
069         * 
070         * @return
071         */
072        public Object getFirstSlotValue() {
073            if (slotValues.size() > 0) {
074                return slotValues.get(0);
075            } else {
076                return null;
077            }
078        }
079    
080        /**
081         * Set the first slot value. If there is already a slotValue(s) in the list, then reset the value, else add a slotValue.
082         * 
083         * @param slotValue
084         */
085        public void setFirstSlotValue(Object slotValue) {
086            /*
087             * If there is already a slotValue(s) in the list, then reset the value, else add a slotValue
088             */
089            if (slotValues.size() > 0) {
090                slotValues.set(0, slotValue);
091            } else {
092                addSlotValue(slotValue);
093            }
094        }
095    
096        /**
097         * Add a slot value to this slot mention
098         * 
099         * @param slotValue
100         */
101        public void addSlotValue(Object slotValue) {
102            this.slotValues.add(slotValue);
103        }
104    
105        /**
106         * Return a string representation of this SlotMention in a single line.<br>
107         * Representation: " [mentionName]:value1:value2:value3..."
108         * 
109         * @return
110         */
111        @Override
112        public String getSingleLineRepresentation() {
113            String repStr = " [" + getMentionName() + "]:";
114    
115            for (Object object : getSlotValues()) {
116                repStr += object.toString() + ":";
117            }
118            repStr = repStr.substring(0, repStr.length() - 1);
119            return repStr;
120        }
121    
122        public String getDocumentLevelSingleLineRepresentation() {
123            String repStr = " [" + getMentionName() + "]:";
124    
125            for (Object object : getSlotValues()) {
126                repStr += object.toString() + ":";
127            }
128            repStr = repStr.substring(0, repStr.length() - 1);
129            return repStr;
130        }
131        
132        /**
133         * Output a description of this slot mention
134         * 
135         * @param sm
136         * @param ps
137         * @param indentLevel -
138         *            controls the size of the indent from the left
139         */
140        public static void printMention(SlotMention sm, PrintStream ps, int indentLevel) {
141            String indentSpace = "-";
142            for (int i = 0; i < indentLevel * 4; i++) {
143                indentSpace += " ";
144            }
145            ps.print(indentSpace + "SLOT MENTION: " + sm.getMentionName() + " with SLOT VALUE(s): ");
146            for (Object value : sm.getSlotValues()) {
147                ps.print(value + "  ");
148            }
149            ps.println();
150        }
151    
152        /**
153         * 
154         * @return
155         */
156        @Deprecated
157        public String getHashKey() {
158            String key = this.getMentionName();
159            for (Object obj : slotValues) {
160                key += (" " + obj);
161            }
162            return key;
163        }
164    
165        /**
166         * Compares two slot mentions by comparing their single line representations. By default we use the IdenticalSlotMentionComparator
167         */
168        public int compareTo(Object o) {
169            if (o instanceof SlotMention) {
170                SlotMention smToCompare = (SlotMention) o;
171                IdenticalSlotMentionComparator ismc = new IdenticalSlotMentionComparator();
172                return ismc.compare(this, smToCompare);
173                // return getSingleLineRepresentation().compareTo(compareSM.getSingleLineRepresentation());
174            } else {
175                System.err.println("Unexpected object when comparing to SlotMention: object = " + o.getClass().getName());
176                return -1;
177            }
178        }
179    
180        /**
181         * Two slot mentions are equal if their slot mention name is equal, and their slot values are equal
182         */
183        @Override
184        public boolean equals(Object obj) {
185            if (obj instanceof SlotMention) {
186                SlotMention sm = (SlotMention) obj;
187                /* There will be no text annotations linked to a slot mention, so null is inserted in place of a SpanComparator  */
188                return this.equals(sm, new IdenticalSlotMentionComparator(), null, -1, 0);
189            } else {
190                System.err.println("Cannot directly compare a SlotMention to " + obj.getClass().getName());
191                return false;
192            }
193        }
194    
195        public boolean equals(SlotMention smToCompare, SlotMentionComparator slotMentionComparator, SpanComparator spanComparator,int maximumComparisonDepth, int depth
196                ) {
197            if (slotMentionComparator.compare(this, smToCompare, spanComparator, maximumComparisonDepth, depth) == 0) {
198                return true;
199            } else {
200                return false;
201            }
202        }
203    
204        // @Override
205        // /** Two slot mentions are equal if their slot mention name is equal, and their slot values are equal */
206        // public boolean equals(Object obj) {
207        // if (obj instanceof SlotMention) {
208        // SlotMention sm = (SlotMention) obj;
209        // if (this.getSlotValues().size() == sm.getSlotValues().size()) {
210        // /* quick check to make sure each slot mention has the same number of slot values */
211        // if (this.getMentionName().toLowerCase().equals(sm.getMentionName().toLowerCase())) {
212        // for (Object slotValue : this.getSlotValues()) {
213        // boolean objectHasMatch = false;
214        // for (Object svToCompare : sm.getSlotValues()) {
215        // if (slotValue.equals(svToCompare)) {
216        // objectHasMatch = true;
217        // }
218        // }
219        // if (!objectHasMatch) {
220        // return false;
221        // }
222        // }
223        // /* if we get to this point, then all slot values have a match, so return true */
224        // return true;
225        // } else {
226        // /* the sm's have different mention names */
227        // return false;
228        // }
229        // } else {
230        // /* the sm's have unequal number of slot values */
231        // return false;
232        // }
233        // } else {
234        // System.err.println("Cannot directly compare a SlotMention to " + obj.getClass().getName());
235        // return false;
236        // }
237        // }
238    
239        @Override
240        public int hashCode() {
241            return getSingleLineRepresentation().hashCode();
242        }
243    
244    }
```
